# Supplementary material for: Avoiding ecosystem and social impacts of hydropower, wind, and solar in Southern Africa’s low-carbon electricity system
Source: Nat Commun. 2024 Feb 5;15:1083. doi: 10.1038/s41467-024-45313-z (PMC10844333; doi:10.1038/s41467-024-45313-z)
Supplement: Supplementary file 1 — Supplementary Information [file 41467_2024_45313_MOESM1_ESM.pdf]

Supplementary Information

Avoiding ecosystem and social impacts of hydropower, wind, and solar in Southern Africa's low-carbon electricity system

Grace C. Wu[1,*], Ranjit Deshmukh[1,2,*], Anne Trainor[3], Anagha Uppal[4], AFM Kamal Chowdhury[1,5], Carlos Baez[4], Erik Martin[4], Jonathan Higgins[4], Ana Mileva[6], Kudakwashe Ndhlukula[7]

[1] Environmental Studies, Bren Hall, University of California Santa Barbara, CA 93106
[2] Bren School of Environmental Science and Management, University of California, Santa Barbara, United States
[3] Africa Program, The Nature Conservancy, Arlington, VA, 22203, USA
[4] Department of Geography, Ellison Hall, University of California, Santa Barbara, Santa Barbara, CA 93106, USA
[5] Earth System Science Interdisciplinary Center, University of Maryland, College Park, United States
[6] North America Science Team, The Nature Conservancy, Arlington, VA, 22203, USA
[7] Global Freshwater Team, The Nature Conservancy, Arlington, VA, 22203, USA
[8] Blue Marble Analytics, San Francisco, United States
[9] SADC Centre for Renewable Energy and Energy Efficiency (SACREEE), 11 Dr Agostinho Neto Road, Windhoek, Namibia
[*]Contributed equally. Corresponding authors; email addresses: gracecwu@ucsb.edu (Grace C. Wu), rdeshmukh@ucsb.edu (Ranjit Deshmukh)

This PDF file includes:

- Supplementary Tables 1 to 4
- Supplementary Figures 1 to 8

Supplementary Table 1. Exclusion tiers

Tier	Name	Datasets included
A	Legally protected areas	• International Union for Conservation of Nature (IUCN) categories I and II (wilderness areas and national parks)<br>• Including specific nationally designated areas with legal protections against development
B	Socially important areas	• World heritage sites, catchment areas, communal or community conservancies, community reserve, forest plantations, forest conservancies, community forests, communal land including country-specific datasets<br>• Rangelands (where more than 80% of the area is rangelands) for wind only<br>• All croplands for solar and only croplands with tree or shrub cover for wind
C	Environmentally important areas	• IUCN Categories III (natural monument or feature), IV (habitat or species management area), V (protected landscape or seascape), VI (protected area with sustainable use of natural resources);<br>• Ramsar sites, key biodiversity areas, conservation areas from national government sources, forest and nature reserves
D	Important landscapes	• Intact forests (areas with more than 15% tree cover)<br>• Wetlands

Supplementary Table 2. Scenarios

Scenario name	Tiers excluded	
	Wind and solar	Hydropower
Base	Tier A	None (all planned and proposed projects)
Legal	Tier A	Tier A
Social	Tier A + B	Tier A + B
Environmental	Tier A + C	Tier A + C + large free flowing rivers
Environmental and Landscape	Tier A + C + D	Tier A + C + large free flowing rivers + D
All Exclusions	Tier A + B + C + D	Tier A + B + C + D + large free flowing rivers
All Exclusions No New Hydro	Tier A + B + C + D	All planned/proposed hydropower excluded

Supplementary Table 3. Data sources

See Excel table linked here:
https://docs.google.com/spreadsheets/d/147ZRXg2OVYpuIqzNjT3f0_O6gXdIvRTh?rtpof=true&usp=drive_fs
Also uploaded to Figshare repository.

Supplementary Table 4: Inventory of planned and existing hydropower projects, scenario screening, and results for each scenario

See Excel table linked here for low carbon capped scenarios:
https://drive.google.com/open?id=14NLjaPJUv5U14fFJE2NHId7dQbhLcFEF&usp=drive_fs
See Excel table linked here for normal/no carbon capped scenarios:
https://drive.google.com/file/d/1DPOqtFwdkW8cvTfwhYN-hKNG52meNZjt/view?usp=sharing
(both files are also available on Figshare repository)

Each csv contains the following columns:

Column name	Explanation
id	Unique ID
Project name	Name of hydropower project
Country	Country
River basin	Major river basin
Base scenario	Project status for each scenario:<br>• Existing (not a model choice)<br>• Selected (i.e., chosen as economically competitive by the model)<br>• Suitable NonSelected (it was made available to the model in this scenario, but was not chosen)<br>• Unsuitable Project (it was not made available to the model in this scenario due to being unsuitable)<br>• PH (pumped hydropower)
Legal	
Environmental scenario	
Environmental and Landscape scenario	
Social	
All Exclusions scenario	
Reservoir Volume (m3)	Modeled reservoir volume
Reservoir Area (m2)	Modeled reservoir area
Shp_Lng	Modeled reservoir length in meters
Shap_Ar	Modeled reservoir length in meters
Hydraulic head (m)	Modeled reservoir area in sq meters
Adjusted dam height (m)	Dam height in meters
Reservoir modeled	Y (Yes) or N (No)
Overlap_TierA_Legal_km2	Overlap of reservoir with Tier A datasets (legal) in sq km
Overlap_TierC_Env_km2	Overlap of reservoir with Tier C datasets (environmental) in sq km
Overlap_Rangelands_km2	Overlap of reservoir with Rangelands (in Tier B, social) in sq km
Overlap_Forested_km2	Overlap of reservoir with Forested (in Tier D, landscape) in sq km
Overlap_Cropland_km2	Overlap of reservoir with Croplands (in Tier B, social) in sq km
Human population (persons)	Number of people living within the area of the modeled reservoir
Status	Project status, one of: Existing, committed, candidate (from the SAPP master plan)
Latitude	Location (latitude) in decimal degrees
Longitude	Location (longitude) in decimal degrees

Column name	Explanation
Model group	Whether the project was modeled as its own variable, or grouped with (an)other project(s) as a variable.
Capacity (MW)	Nameplate capacity in megawatts
Capacity_factor	Capacity factor estimated using VIC-RES
Notes	Notes justifying the screening
Overlap_TierA_legal_percent	Percent of reservoir area that overlaps with Tier A datasets (legal) in sq km
Overlap_TierC_Env_percent	Percent of reservoir area that overlaps with Tier C datasets (environmental) in sq km
Overlap_Rangelands_percent	Percent of reservoir area that overlaps with Rangelands (in Tier B, social) in sq km
Overlap_Forested_percent	Percent of reservoir area that overlaps with Forested (in Tier D, landscape) in sq km
Overlap_Cropland_percent	Percent of reservoir area that overlaps with Croplands (in Tier B, social)
Tier A (Legal) screen	Whether or not it passed or failed the Legal screen. Existing means it was not screened.
Tier A (Legal) screen comment	Comments to support legal screen outcome
EnvironmentalTier	Whether or not it passed or failed the Environmental (Tier C) screen. Existing means it was not screened.
Large free flowing river screen	Whether or not it passed or failed the large free flowing river screen. Existing means it was not screened.
Large free flowing river screen comments	Comments to support large free flowing screen outcome
Any free flowing river screen	Whether or not it passed or failed the any free flowing river screen. Existing means it was not screened.
Any free flowing river screen comments	Comments to support any free flowing screen outcome

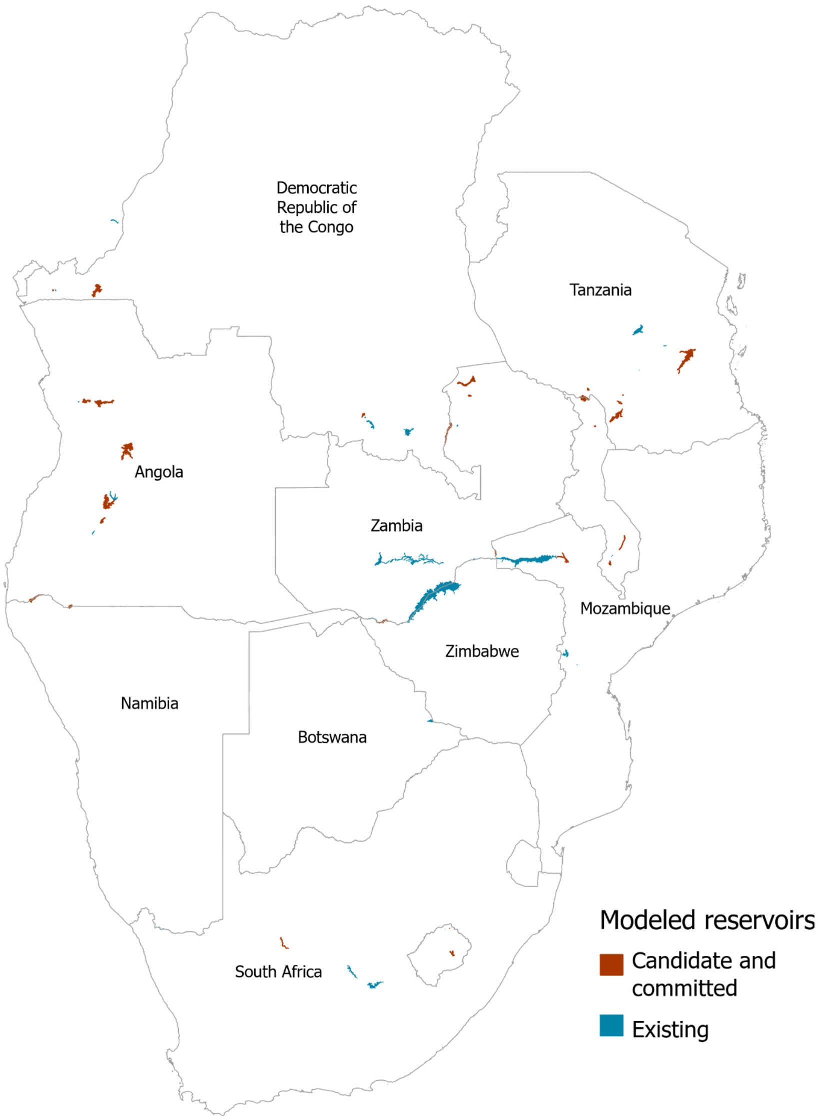

Supplementary Figure 1: Modeled reservoirs for candidate/committed hydropower projects. Dam locations, dam height, digital elevation model, and hydrological watershed and river flow data were used to estimate reservoir area and volume. A select number of existing reservoirs were modeled to validate the approach.

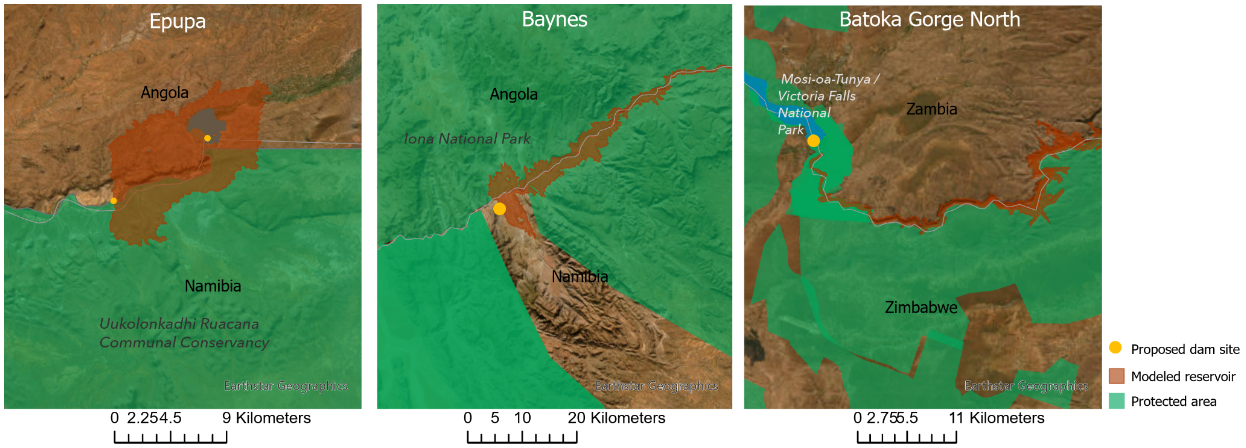

Supplementary Figure 2: Examples of screened hydropower projects and their modeled reservoirs. Both Baynes and Batoka Gorge North were screened out in the Legal scenario due to overlap with national parks, and Epupa was screened out in the Social scenario due to overlap with a community land conservancy in Namibia.

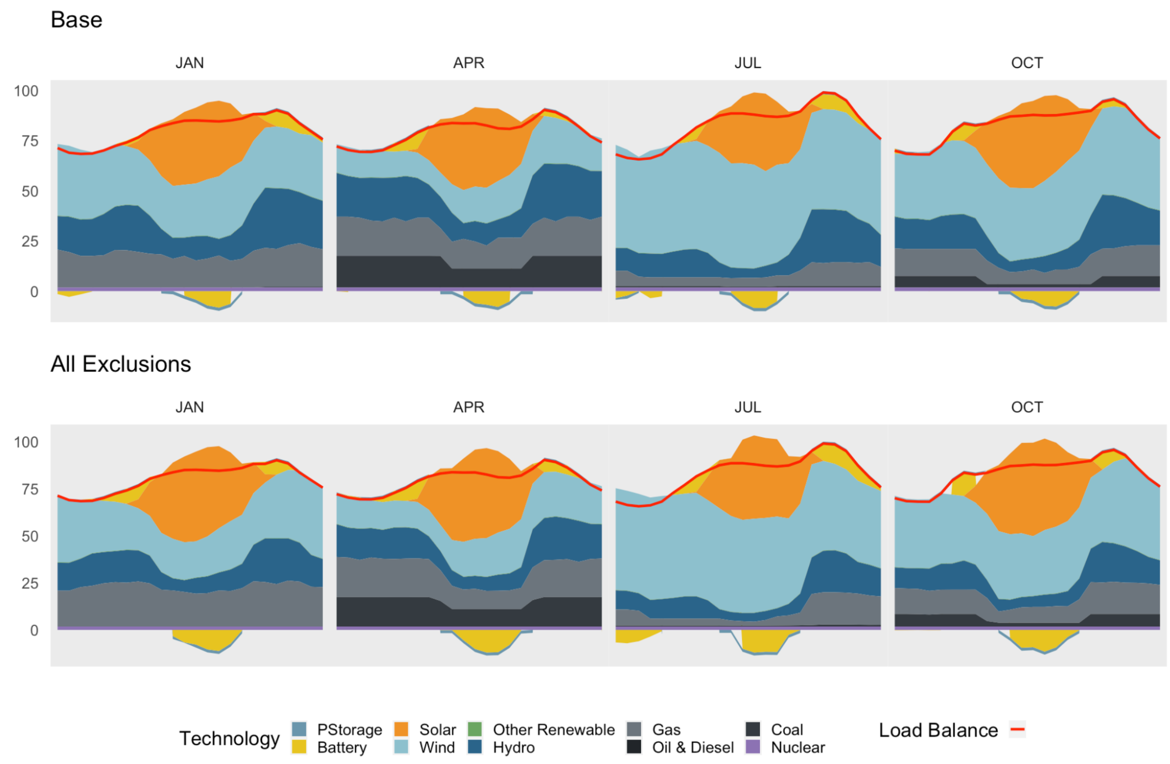

Supplementary Figure 3: Hourly dispatch for representative days and months in 2040. Each panel shows the hourly dispatch for each generation or storage technology for a representative day in January (JAN), April (APR), July (JUL), and October (OCT). PStorage refers to pumped storage.

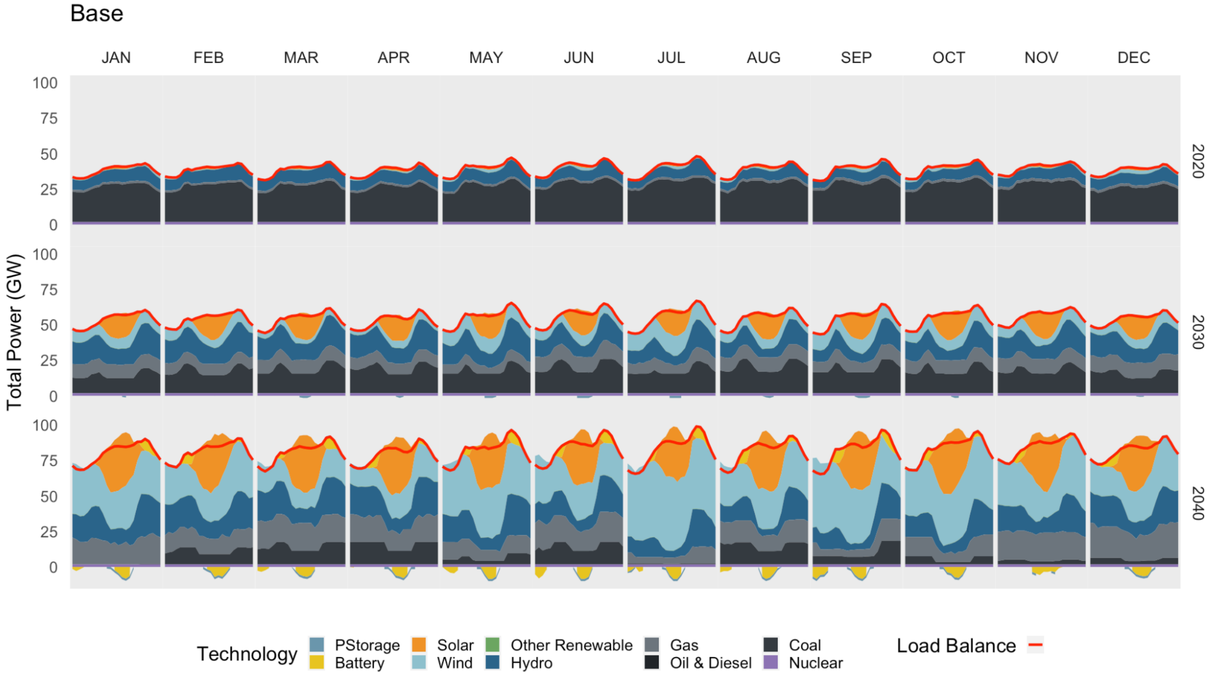

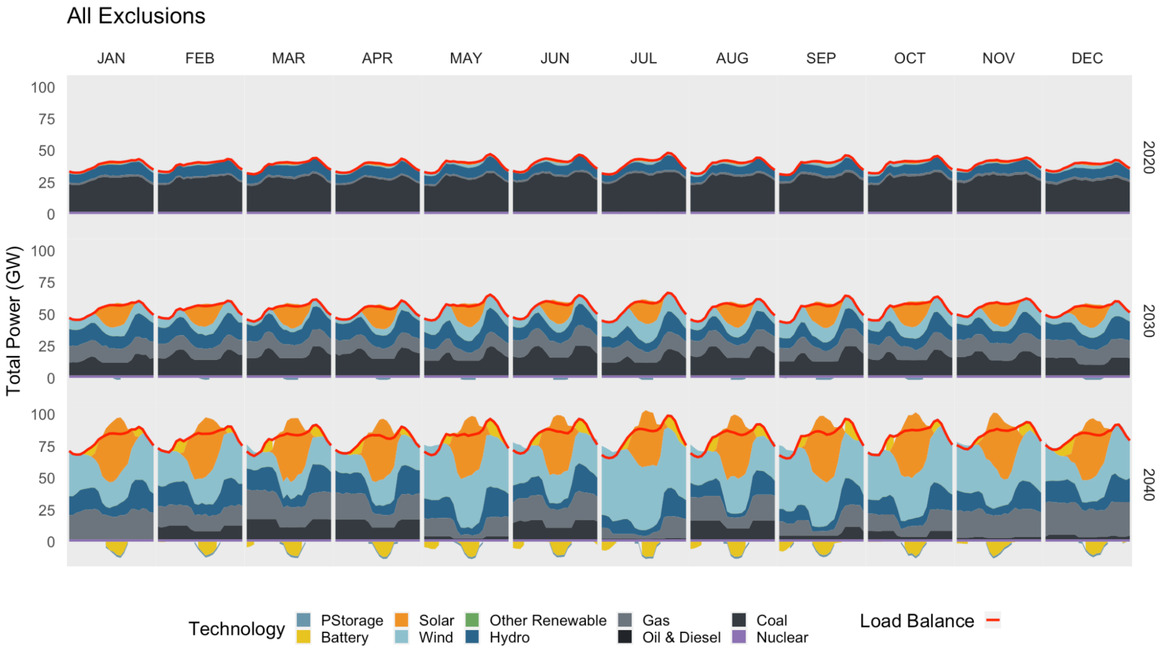

Supplementary Figure 4: Hourly dispatch for representative days and all months for 2020, 2030, and 2040. The top panel for the *Base* scenario and the bottom panel is for the *All Exclusions* scenario. PStorage refers to pumped storage.

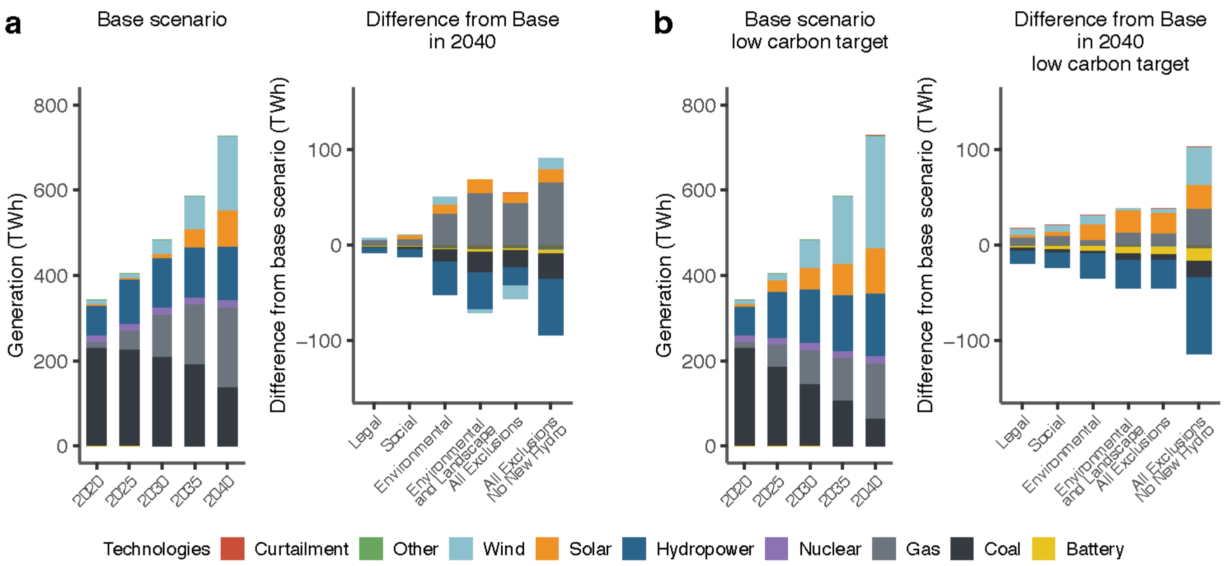

Supplementary Figure 5: Generation mix of scenarios with and without a carbon target. (a) Generation by technology from 2020-2040 for the Base scenario without a carbon target and differences in generation in 2040 for each scenario compared to *Base*. (b) Same as (a) but with a low carbon emissions target trajectory that limits annual carbon emissions in 2040 to half of carbon emissions in 2020. Positive differences indicate more generation and negative differences indicate less generation compared to the *Base* scenarios. Source data are provided as a Source Data file.

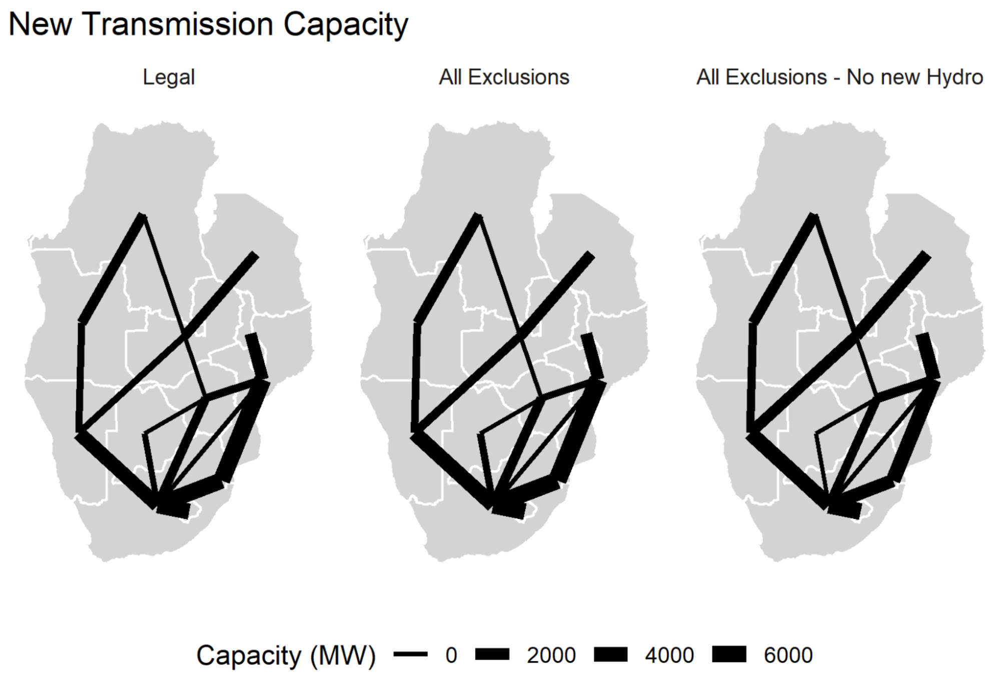

Supplementary Figure 6: Transmission flows between countries. Thickness lines correspond to the selected transmission capacity in each scenario. See Figure S1 for country name labels.

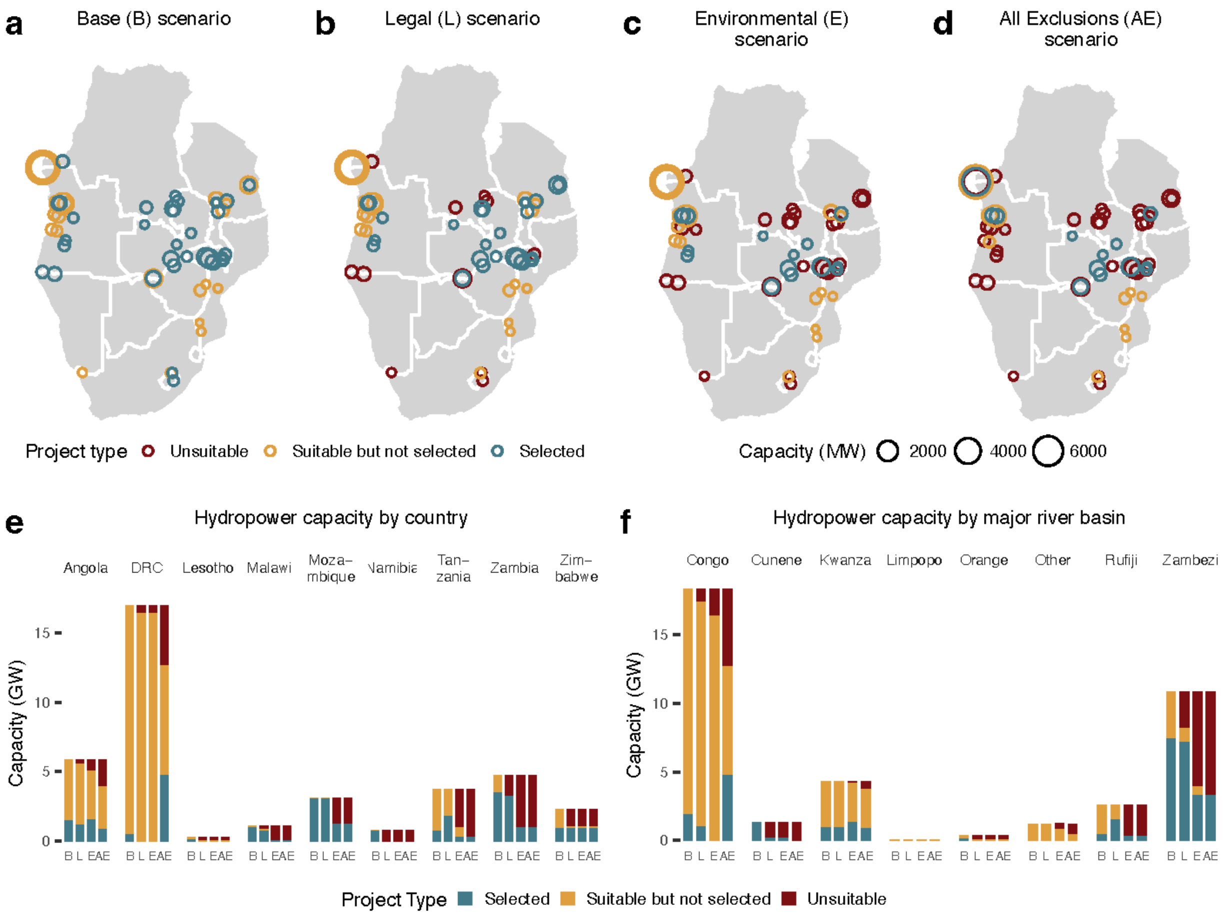

Supplementary Figure 7: Selected hydropower capacity in the scenarios without a carbon target. Spatial distribution (a, b, c), country-wise capacities (d), and basin-wise capacities (e) of unsuitable, suitable but not selected, and selected hydropower projects for Base (B), Legal (L), Environmental (E), and All Exclusion (AE) scenarios for the no-carbon target case. Source data are provided as a Source Data file. See Figure S1 for country name labels.

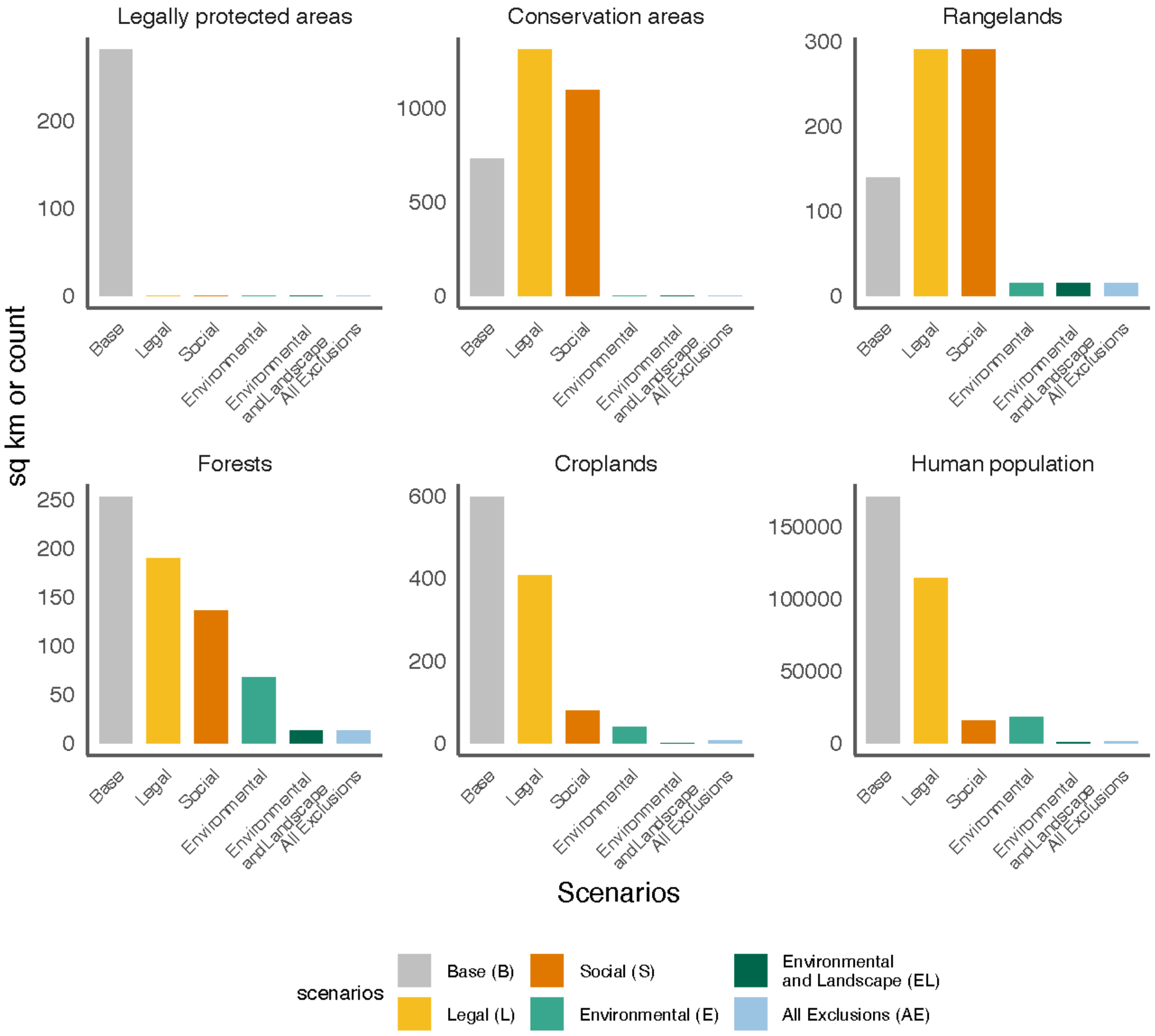

Supplementary Figure 8: Environmental and social impacts of selected hydropower plants in the scenarios without a carbon target. Environmental and social impacts (in square kilometers of inundated area or number of people displaced) of selected hydropower projects for scenarios meeting the low-carbon target. Source data are provided as a Source Data file.
